# Comment on case of benralizumab‐induced exacerbations of chronic spontaneous urticaria

**DOI:** 10.1002/ccr3.8802

**Published:** 2024-04-17

**Authors:** Mustafa Ilker Inan, Yasemin Akgul Balaban

**Affiliations:** ^1^ Division of Immunology and Allergic Diseases Ankara Gulhane Training And Research Hospital Ankara Turkey

## CONSENT

This is a letter for a case report that was published in your journal, so the patient was not our patient and we were unable to obtain a consent form.

Dear Editor,

In the 10th volume, 6th issue of Clinical Case Reports, we read the article of Dr Megan and colleagues entitled “Case of benralizumab‐induced exacerbations of chronic spontaneous urticaria.”^1^ We thank for their valuable case report which they mentioned about benralizumab induced urticaria. In this case report they gave benralizumab treatment to an uncontrolled severe asthma patient who had an history of autologous serum skin test positive chronic spontaneous urticaria (CSU). Although this is a straightforward and well‐designed study, we want to mention about some points.

The authors stated that their patient had nonseasonal allergic rhinitis and atopic asthma for 18 years, but did not mention which inhalant allergen sensitisation the patient had and the level of serum total Ig E.

It was reported that the patient's asthma was poorly controlled with budesonide‐formoterol (bid 320/9 μg) + montelukast 10 mg + prednisone 40 mg treatments, but the given inhaled budesonide dose was not sufficient to be considered a high dose.^2^

The role of biologic agents in the treatment of severe asthma has been increasing, recently. The current biologic agents available nowadays are targeting IgE, IL‐5, IL‐4/IL‐13, and TSLP.^3^ The authors did not explain why they chose benralizumab treatment as the first choice, although other biologic agent treatment options are available. Omalizumab is an anti‐IgE monoclonal antibody. It is approved for treatment of allergic asthma in individuals who have evidence of sensitivity to perennial allergens and IgE levels between 30 and 700 kU/L.^4^ In addition, in the treatment of CSU, the use of omalizumab is recommended in patients with antihistamine‐resistant disease.^5^ Mepolizumab and dupilumab are humanized monoclonal antibodies targeting IL‐5 and IL4 receptor‐α, respectively. They are effective in individuals with eosinophilic asthma defined as having peripheral blood eosinophil counts of at least 150 cells/μL.^4^, ^6^

Although total Ig E level was not mentioned, we think that omalizumab treatment would be more appropriate as the first choice in this patient with a history of perennial allergic rhinitis and CSU.

Switching between biological agents may be considered in cases of inadequate response to current treatment and/or any adverse event develops.^3^ Although the patient's urticaria‐angioedema clinic increased after benralizumab treatment, they did not explain why they still insisted on benralizumab treatment and restarted it without considering to switch other alternative biologic agents.

In case of any adverse drug reaction, the first step of treatment is stopping the culprit agent. Instead of using the culprit drug, a safe alternative drug options should be offered. If alternative treatment options are not sufficient and/or effective, desensitization may be considered.^7^, ^8^

As a result, we think that it is unrealistic to prefer benralizumab as first‐line treatment and to insist on benralizumab despite the development of side effects. Clarifying these concerns will provide clearer picture to the readers.

## AUTHOR CONTRIBUTIONS

**Mustafa Ilker Inan:** Investigation; methodology; resources; software; validation; writing – original draft; writing – review and editing. **Yasemin Akgul Balaban:** Investigation; writing – original draft; writing – review and editing.

## CONFLICT OF INTEREST STATEMENT

None.

## Data Availability

The data that support the findings of this study are available from the corresponding author upon reasonable request option.
